# Frontline Science: Endotoxin‐induced immunotolerance is associated with loss of monocyte metabolic plasticity and reduction of oxidative burst

**DOI:** 10.1002/JLB.5HI0119-018R

**Published:** 2019-06-06

**Authors:** Inge Grondman, Rob J. W. Arts, Rebecca M. Koch, Guus P. Leijte, Jelle Gerretsen, Niklas Bruse, Rosalie W. M. Kempkes, Rob ter Horst, Matthijs Kox, Peter Pickkers, Mihai G. Netea, Mark S. Gresnigt

**Affiliations:** ^1^ Department of Internal Medicine Radboud University Medical Center Nijmegen the Netherlands; ^2^ Radboud Center for Infectious Diseases Radboud University Medical Center Nijmegen the Netherlands; ^3^ Department of Intensive Care Medicine Radboud University Medical Center Nijmegen the Netherlands; ^4^ Department for Genomics & Immunoregulation Life and Medical Sciences Institute (LIMES) University of Bonn Bonn Germany; ^5^ Department of Microbial Pathogenicity Mechanisms Leibniz Institute for Natural Product Research and Infection Biology, Hans Knöll Institute Jena Germany

**Keywords:** endotoxemia, endotoxin tolerance, immunometabolism, immunoparalysis, monocytes, sepsis

## Abstract

Secondary infections are a major complication of sepsis and associated with a compromised immune state, called sepsis‐induced immunoparalysis. Molecular mechanisms causing immunoparalysis remain unclear; however, changes in cellular metabolism of leukocytes have been linked to immunoparalysis. We investigated the relation of metabolic changes to antimicrobial monocyte functions in endotoxin‐induced immunotolerance, as a model for sepsis‐induced immunoparalysis. In this study, immunotolerance was induced in healthy males by intravenous endotoxin (2 ng/kg, derived from *Escherichia coli* O:113) administration. Before and after induction of immunotolerance, circulating CD14^+^ monocytes were isolated and assessed for antimicrobial functions, including cytokine production, oxidative burst, and microbial (*Candida albicans*) killing capacity, as well metabolic responses to ex vivo stimulation. Next, the effects of altered cellular metabolism on monocyte functions were validated in vitro. Ex vivo lipopolysaccharide stimulation induced an extensive rewiring of metabolism in naive monocytes. In contrast, endotoxin‐induced immunotolerant monocytes showed no metabolic plasticity, as they were unable to adapt their metabolism or mount cytokine and oxidative responses. Validation experiments showed that modulation of metabolic pathways, affected by immunotolerance, influenced monocyte cytokine production, oxidative burst, and microbial (*C. albicans*) killing in naive monocytes. Collectively, these data demonstrate that immunotolerant monocytes are characterized by a loss of metabolic plasticity and these metabolic defects impact antimicrobial monocyte immune functions. Further, these findings support that the changed cellular metabolism of immunotolerant monocytes might reveal novel therapeutic targets to reverse sepsis‐induced immunoparalysis.

## Graphical abstract

Endotoxin‐induced immunotolerant monocytes are characterized by a loss of metabolic plasticity and these metabolic defects impact antimicrobial monocyte immune functions.

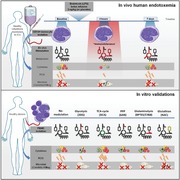

**Abbreviations**
2DG: 2‐deoxy‐d‐glucose
6AN: 6‐aminonicotinamide
AUC: area under the curve
BPTES bis‐2‐(5‐phenylacetamido‐ 1,3,4‐: bis‐2‐(5‐phenylacetamido‐ 1,3,4‐thiadiazol‐2‐yl)ethyl sulfide
BSO: buthionine sulfoximine
C75: 4‐methylene‐2‐octyl‐5‐oxotetrahydrofuran‐3‐carboxylic acid
CFU: colony forming unit
DCA: potassium dichloroacetate
DPI: diphenyleneiodonium
ETO: Etomoxir
ICU: Intensive care unit
NAC: *N*‐acetyl‐l‐cysteine
OLI: Oligomycin
Ox‐Phos: Oxidative phosphorylation
PCA: principal component analysis
PDC: pyruvate dehydrogenase complex
PPP: pentose phosphate pathway
‐PYR: without pyruvate
ROS: reactive oxygen species
SOX: sodium oxamate

## 1 INTRODUCTION

Secondary infections represent a major complication for patients admitted to the intensive care unit (ICU) with sepsis.^1^ In sepsis, hyperinflammatory responses to infection are accompanied and/or followed by counterregulatory anti‐inflammatory responses, which can cause a dysfunctional state in which immune cells are unable to adequately respond to pathogens.^2^ This phenomenon, called sepsis‐induced immunoparalysis, is associated with increased susceptibility to secondary (opportunistic) infections.^3^ Microbial infections in the late phase of sepsis including bacteria (e.g., *Escherichia coli* and *Staphylococcus aureus*), fungi (e.g., *Candida albicans*), and viral reactivation all illustrate the clinical significance of sepsis‐induced immunoparalysis, which is associated with increased ICU length of stay.^4^, ^5^, ^6^ Moreover, the dysregulated host response is considered to be a critical factor that contributes to the persistently high mortality in sepsis, as a result of secondary infections.^7^, ^8^

Monocytes are severely affected during immunoparalysis, and they play a pivotal role in the host response during sepsis. A low human leukocyte antigen–antigen D related (HLA‐DR) expression on monocytes coincides with an increase in nosocomial infections and higher mortality.^9^, ^10^ Another hallmark of sepsis‐induced immunoparalysis are impaired ex vivo monocyte cytokine responses, which are likely a result of cellular reprogramming.^11^ Therefore, reversing or preventing monocyte immunotolerance represents an attractive strategy to improve immune function and outcome of sepsis.^12^ To facilitate the development of effective therapies, the molecular mechanisms underlying the immunotolerant state of monocytes should be unraveled.

In the past decade, it has become clear that during sepsis immune function and cellular metabolism are closely interlinked.^13^ A previous study revealed dysfunction of cellular metabolism in immunotolerant monocytes.^14^ Recently, in vitro LPS tolerization was found to induce prominent metabolic changes over time in THP‐1 monocyte cells.^15^ This study demonstrated a consecutive metabolic adaptation of these cells during immune activation, deactivation (tolerance), and resolution of inflammation. These findings support the notion that metabolic rewiring after immune stimulation may be a major mechanism that drives the tolerant state of immune cells. Importantly, this study highlights the need for in vivo studies to further characterize metabolic changes in tolerant immune cells and the impact on immune function in parallel with this metabolic shift. A clear understanding of the essential metabolic routes affected during immunoparalysis and how they interfere with immune function is still lacking. This knowledge gap needs to be bridged before effective therapies can be developed.

In the current study, the primary aim was to better understand the potentially defective adaptation of monocyte metabolic responses during immunoparalysis. To this end, a standardized protocol^16^ of endotoxin‐induced immunotolerance was employed, where human subjects were intravenously challenged with endotoxin (*E. coli* LPS) to model sepsis‐induced immunoparalysis. Monocytes were studied before, during, and 1 week after endotoxin‐induced immunotolerance to characterize metabolic changes associated with immunotolerance and long‐lasting metabolic reprogramming following endotoxin challenge. First, the metabolic profile and immunological functions were characterized ex vivo before immunotolerance, during immunotolerance and 1 week after immunotolerance. Second, metabolic pathways found to be affected were validated for their impact on antimicrobial functions in naive monocytes.

## 2 MATERIALS AND METHODS

### 2.1 Experimental human endotoxemia model

Experimental human endotoxemia was used as a model for sepsis‐induced immunoparalysis, as extensively described in previously published studies.^16^, ^17^ After written informed consent was obtained, blood samples from healthy male volunteers were collected. Subjects participated in two similarly designed endotoxemia studies (NL54870.091.15;^17^ NL61136.091.16 [unpublished data]), both approved by the local medical ethics committee. Male healthy volunteers aged 18–35 years, all Caucasian, were screened at the Department of Intensive Care Medicine of the Radboud University Medical Center, Nijmegen, the Netherlands. Screening included a normal physical examination, electrocardiography, and routine laboratory values (including serology on HIV and hepatitis B). Exclusion criteria included febrile illness 2 weeks before baseline, a suspicion of influenza infection in the preceding year, pre‐existent (lung) disease, recent vaccination, and usage of any prescription drugs. Subjects refrained from caffeine and alcohol 24 h before experiments and from food 12 h before experiments, as well as during experiments. In total 30 healthy volunteers were included in the first study (NL54870.091.15).^17^ Of these, 15 of volunteers were assigned to receive placebo and 15 were assigned to the endotoxin infusion group. In the second endotoxin study (NL61136.091.16 [unpublished data]), a total of 12 healthy volunteers were included. Of these, 4 volunteers were in the placebo group and 8 were in the endotoxin infusion group. Participants were hospitalized at the Intensive Care Research Unit of the Radboud University Medical Center, and all study‐related procedures were assessed according to the principles of the most recent revised Declaration of Helsinki. They received either endotoxin (intravenous bolus administration of 2 ng/kg LPS; U.S. Standard Reference Endotoxin derived from *E. coli* O:113, Pharmaceutical Development Section of the National Institutes of Health, Bethesda, MD, USA) or placebo (0.9% saline). From the original study of 30 volunteers (NL54870.091.15),^17^ we obtained blood samples at baseline (BL), 4 hours (4h), and 7 days (7d) following endotoxin or placebo administration from 6 placebo controls and 15 volunteers receiving endotoxin. All these samples were analyzed for cytokine responses, and monocyte samples from 5 randomly selected volunteers receiving endotoxin were selected for metabolic analysis. In the second study, blood samples were similarly obtained at all aforementioned time points. Blood samples from 4 placebo and 6 endotoxin challenged subjects were available for analysis of oxidative burst. Parallel, samples from the 5 volunteers receiving endotoxin in this study were analyzed for intracellular killing capacity. The time points were selected based on previous in vivo endotoxemia studies that demonstrated that 4 h following endotoxin administration monocytes demonstrate an immunotolerant phenotype.^16^, ^18^, ^19^ The 7‐day time point was selected to investigate whether metabolic changes associated with immunotolerance are persisting over more extended periods, maybe even after the immunological tolerance is restored.

### 2.2 Peripheral blood mononuclear cells and CD14+ monocyte isolation

Blood was diluted (1:1) in PBS. PBMCs were isolated from blood by differential density gradient centrifugation over Ficoll‐Paque PLUS (protocol supplied by GE Healthcare, Chicago, IL, USA). Cells were washed twice in PBS and resuspended in RPMI 1640 culture medium Dutch modification (Gibco; Thermo Fisher, Waltham, MA, USA), supplemented with pyruvate (1 mM; Gibco), glutamax (2 mM; Gibco), and gentamicin (50 μg/ml; Centrafarm, Etten‐Leur, the Netherlands). Classical monocytes were subsequently isolated from the PBMC fraction using magnetic beads labeled with anti‐CD14 (protocol: MACS Miltenyi, Bergisch Gladbach, Germany). Finally, cells were counted using the Sysmex XN‐450 automated differential hematology analyzer (Sysmex Corporation, Kobe, Japan).

### 2.3 Ex vivo experiments

Isolated CD14+ monocytes were plated in 96‐well tissue treated flat‐bottom plates (1.0 × 10^5^ cells/well; Eppendorf, Hamburg, Germany) and stimulated with RPMI or *E. coli* LPS (10 ng/ml; serotype 055:B5, Sigma‐Aldrich) for 24 h at 37°C and 5% CO_2_. Monocyte cytokine responses were measured in supernatants of 15 endotoxin‐challenged subjects and 6 placebo subjects.

#### 2.3.1 Monocyte metabolic profiling

Metabolic profiling was performed on ex vivo LPS‐restimulated and unstimulated CD14+ monocytes isolated from 5 randomly selected volunteers that were infused with endotoxin. Samples were placed in sterile micro centrifuge tubes (2 × 10^6^ cells/tube in a volume of 1 ml). Cell pellets were snap frozen in liquid nitrogen and shipped to Metabolon (Morrisville, USA). Quantitative measurements of metabolite concentrations were assessed in classical monocytes at baseline, 4 h, and 7 days following endotoxemia. Metabolon performed the identification and quantification of amino acids, carbohydrates, lipids, nucleotides, microbiota metabolism, energy metabolism, cofactors and vitamins, xenobiotics, and novel metabolites.

#### 2.3.2 Ex vivo monocyte oxidative capacity

In the NL61136.091.16 endotoxemia cohort, oxidative burst capacity was determined in isolated CD14+ of 10 male volunteers (*n* = 6 endotoxemia challenged, *n* = 4 placebo). For these experiments, the total production of reactive oxygen species (ROS) generated by monocytes was measured in flat‐bottomed white tissue‐culture treated 96 well plates (Sigma‐Aldrich, Saint Louis, MO, USA) To every well, 20 μl of 3‐aminophthalhydrazide, 5‐amino‐2,3‐dihydro‐1,4‐phthalazinedione (luminol; 1 mM in DMSO; Sigma‐Aldrich) in HBSS (Gibco) and 0.5% BSA (Sigma‐Aldrich) were added. Monocytes were seeded directly in white 96‐wells plates (1.0 × 10^5^ cells/well) and stimulated with 10% human serum opsonized live *E. coli* (1.0 × 10^7^ bacteria/ml, clinical isolate ATCC35218), *S. aureus* (1.0 × 10^7^ bacteria/ml, clinical isolate ATCC25923), *C. albicans* (1.0 × 10^7^ yeast/ml, strain UC820), or RPMI. In all experiments, the chemiluminescence was measured every minute, at 37°C, by using a BioTek Synergy HT reader (Winooski, VT, USA) for 1 h.

#### 2.3.3 Monocyte *Candida* killing capacity

To determine the intracellular killing capacity of pathogens after experimental endotoxemia, CD14^+^ monocytes (1 × 10^5^ cells/well) of 5 male volunteers were exposed to live *C. albicans* (2 × 10^5^ yeast/well, strain UC820) for 24 h at 37°C and 5%. CO_2_ at baseline, 4 h after endotoxin challenge, and 7 days following endotoxin administration. After incubation, all cells were lysed in water and serial dilutions of lysates were plated on Sabouraud agar plates. The *Candida* killing capacity of CD14+ monocytes was quantified by counting remaining colony‐forming units (CFUs), after overnight incubation at 37°C and 5% CO_2_. The percentage of killed microbes was calculated as 1 − (CFU remained after incubation with microbes/CFU determined before incubation with microbes) × 100.

### 2.4 In vitro model for modulation of metabolic pathways

The local ethical board at the Radboud University Nijmegen (Arnhem‐Nijmegen Medical Ethical Committee) approved blood sampling for the in vitro validation studies using metabolic inhibitors. Venous blood was drawn from healthy volunteers after the healthy volunteers provided written informed consent. The effect of metabolic modulation on immune function was studied in experiments using PBMCs. Briefly, venous blood samples were drawn from healthy volunteers after written informed consent was obtained. PBMCs were isolated by density gradient centrifugation over Ficoll, as described above. Metabolic inhibitors were selected based on the metabolic changes observed during endotoxin‐induced immunotolerance. First, various metabolic pathways were modulated, either inhibited or stimulated, for 24 h. (An overview is presented in Table 1.) Next, cells were assessed for several antimicrobial functions.

**Table 1:** Overview of metabolic modulators used in modulation experiments

Modulator	Target	Metabolic pathway inhibitor/stimulator	Experimental concentration
2DG	Hexokinase (competition for glucose)	Glycolysis (inhibitor)	11 mM
6AN	NADP+‐dependent enzyme, 6‐phosphogluconate dehydrogenase	PPP (inhibitor)	100 μM
SOX	Lactate dehydrogenase (inhibitor)	Pyruvate metabolism (inhibitor)	40 mM
DCA	PDC (stimulates pyruvate entry into the tricarboxylic acid cycle)	Tricarboxylic acid (TCA) cycle (stimulator)	15 mM
OLI	ATP synthase	ATP synthesis and electron transport (mitochondrial) (inhibitor)	1 μM
C75	Fatty‐acid synthase	Fatty acid metabolism (inhibitor)	40 μM
ETO	Carnitine palmitoyltransferase‐1 (CPT‐1) (inhibitor of fatty acid oxidation in mitochondria )	Fatty acid metabolism (inhibitor)	10 μM
NAC	Precursor glutathione	Glutathione synthesis (stimulator)	20 mM
BSO	γ‐Glutamylcysteine synthetase	Glutathione synthesis (inhibitor)	100 μM
BPTES	GLS 1 (glutaminase)	Glutaminolysis (inhibitor)	50 μM
C968	GLS 1 (glutaminase)	Glutaminolysis (inhibitor)	25 μM
DPI	Flavoproteins, in particular NADPH oxidase	NADPH oxidase complex (inhibitor)	10 μM

#### 2.4.1 PBMC stimulation experiments

After 24 h preincubation with modulators, PBMCs (5.0 × 10^5^cells/well) were stimulated with heat‐inactivated *E. coli* (1.0 × 10^7^ bacteria/ml, clinical isolate ATCC35218), *S. aureus* (1.0 × 10^7^/ml, clinical isolate ATCC25923), and *C. albicans* (1.0 × 10^6^/ml, strain UC820) for 24 h. Cytokines were measured in supernatants to assess the effects of metabolic modulation on cytokine responses.

#### 2.4.2 In vitro oxidative burst assays

Similarly, the effects of metabolic modulation on oxidative burst were determined. Supernatants of seeded PBMCs (5.0 × 10^5^ cells/well) were discarded. Subsequently, the PBMCs were stimulated with 200 μl in 10% human serum opsonized live *E. coli* (1.0 × 10^7^ bacteria/ml, ATCC35218), *S. aureus* (1.0 × 10^7^ bacteria/ml, ATCC25923), *C. albicans* (1.0 × 10^6^ yeast/ml, UC820), or RPMI. ROS production was measured with a luminol oxidation assay, similarly as described above for ex vivo experiments.

#### 2.4.3 In vitro NADPH assays

For the detection of intracellular NADPH, isolated PBMCs were plated in 12‐wells flat round‐bottom plates with a density of 4.5 × 10^6^ cells/well. PBMCs were preincubated for 24 h with various modulators, as described before (Table 1). After stimulation for 24 h, stimuli and metabolic modulators were washed away and the intracellular nucleotide NADPH was measured, using Colorimetric (450 nm) Quantification Assay Kit following instructions of the manufacturer (protocol MAK038: Sigma Aldrich, Saint Louis, MO, USA). The absorbance at 450 nm was measured using a BioTek Synergy HT reader (Winooski, VT, USA) at 37°C, after a final incubation time of 120 min at room temperature for the conversion of NADP to NADPH.

#### 2.4.4 In vitro microbial killing assays

After removal of the metabolic modulators, PBMCs were washed twice with warm PBS in order to prevent interference of the metabolic inhibitors and stimuli with the live *C. albicans*. PBMCs were stimulated with RPMI, or *C. albicans* (2 × 10^5^ yeast/well, UC820) for 24 h at 37°C and 5% CO_2_. After incubation, all cells were lysed in water, and serial dilutions of lysates were plated on Sabouraud agar plates (Becton Dickinson, Heidelberg, Germany). The *Candida* killing capacity of the PBMCs was quantified by counting remaining CFUs overnight incubation at 37°C and 5% CO_2_. The killing capacity was calculated as described before.

#### 2.4.5 In vitro viability assays

To determine the effect of the inhibitors and stimuli of specific metabolic pathways on cell viability in PBMCs, apoptosis was assessed using Annexin‐V and propidium iodide (PI) staining. Cells were stained for 15 min in 300 μl RPMI containing 1 μl Annexin‐V (Biovision) and 5 mM CaCl_2_. Immediately before measurement 1.5 μl PI (Invitrogen Molecular Probes) was added. Cells were measured using a CytoFLEX cytometer (Beckman Coulter).

### 2.5 Cytokine measurements

Cytokine measurements of all experiments were performed in collected supernatants that were stored at −20°C until cytokine measurements were performed. TNF‐α, IL‐1β, IL‐1Ra, IL‐10, and IL‐6 levels were measured using commercially available ELISAs according to the protocol supplied by the manufacturer (TNF‐α, IL‐1β, and IL‐1Ra [R&D Systems, Minneapolis, MN, USA], IL‐10 and IL‐6 [Sanquin, Amsterdam, the Netherlands]).

### 2.6 Statistical analysis

The data are represented as the mean ± sem or median, based on their distribution. One‐way ANOVA for repeated measurements was used to compare statistical differences for the means of the respective time points. Dunn's multiple comparison test was used as a post hoc test. The Wilcoxon signed rank test was performed to compare means for significant differences of metabolic modulation in validation experiments. Calculations and statistical analysis were performed with GraphPad Prism Version 5.03 (San Diego, CA, USA.) All computational analyses for metabolome data analysis were performed in the R programming language.^20^ For metabolomics, a pathway analysis was assessed. Each metabolite was assigned to a pathway by Metabolon, and enrichments were calculated for these pathways, comparing two conditions at a time. The conditions that were compared are 4 h (4h) versus baseline, 7 days (7d) versus 4 h (4h), and 7 days (7d) versus baseline. The R package "gage" was used to calculate pathway enrichment, which used log2 metabolite concentrations as an input.^21^ All calculations were performed in a paired fashion, and *P*‐values were calculated using a 2‐sided paired *t*‐test. A Benjamini‐Hochberg false discovery rate procedure was used to correct these *P*‐values for type I errors.^22^ Different sample normalization methods were attempted, including total area normalization, and techniques more robust to outliers like the normalization method available in the DESeq2 package.^23^ In the end, it was found that normalization was not required, since the same amount of sample was used for each measurement, and the values should be semiquantitative. A *P*‐value < 0.05 was considered statistically significant with *P < 0.05, **P < 0.01, and ***P < 0.001.

## 3 RESULTS

### 3.1 Endotoxin‐induced immunotolerance affects monocyte cytokine responses, microbial killing capacity, and oxidative burst

**Figure 1:**
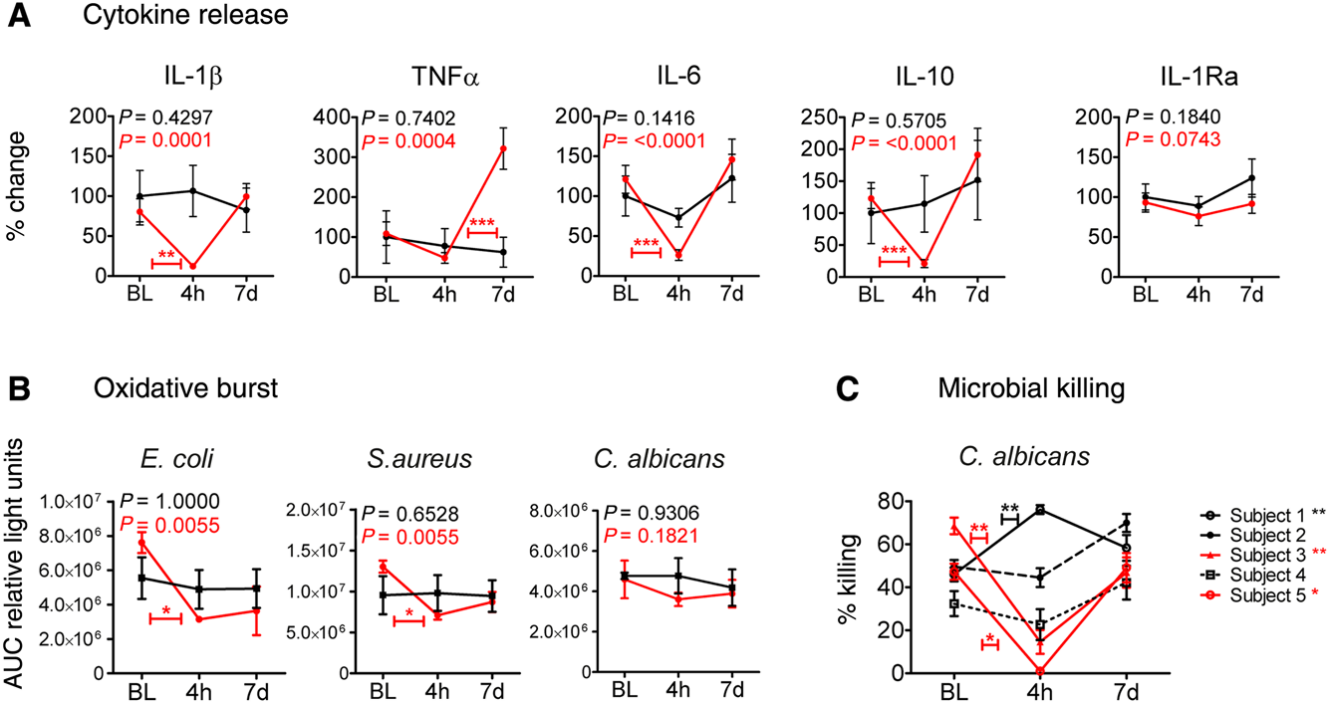
**Endotoxin‐induced monocyte tolerance affects monocyte effector functions**. (**A**) Kinetics of ex vivo LPS‐induced IL‐1β, TNF‐α, IL‐6, IL‐10, and IL‐1Ra levels in culture supernatants of CD14+ monocytes that were isolated from healthy volunteers at baseline (BL), 4 h (4h), and 7 days (7d) after administration of endotoxin (2 ng/kg, red lines, *n *= 15) or placebo (0.9% saline, black lines, *n *= 6). Data are presented as (mean ± sem) percentage change compared to baseline measurements. Differences within groups over time were analyzed using the Friedmann test with Dunn's post hoc test for multiple comparisons. **P < 0.01, ***P < 0.001. (**B**) Kinetics of ex vivo *E. coli*, *S. aureus*, or *C. albicans* induced ROS production (AUC relative light units) in isolated CD14+ monocytes from healthy volunteers for all time points. Red lines present subjects challenged with endotoxin (*n *= 6); black lines show placebo controls (*n* = 4). Data are presented as mean ± sem. Means were compared for significance using the Friedmann test with Dunn's post hoc test to illustrate differences between defined time points. *= *P* < 0.05. (**C**) Ex vivo intracellular killing of *C. albicans* phagocytized by CD14+ monocytes isolated at baseline (BL), 4 h (4h) and 7 days (7d) in (*n *= 5) endotoxin‐challenged subjects, presented for each subject separately. The killing was calculated for duplicates and serial dilutions as 1 – (CFU remained after incubation with microbes/CFU determined before incubation with microbes) × 100%, hence presented as the percentage of *Candida* killing. Data are shown as the mean ± sem. BL versus 4 h was statistically compared for each subject (*=* P* < 0.05, **= *P* < 0.01) with the Mann‐Whitney U test

Intravenous endotoxin administration induces immunotolerance in circulating monocytes in healthy volunteers, and these otherwise highly responsive cells are rendered irresponsive to ex vivo re‐challenge with LPS.^16^, ^18^, ^19^ Accordingly, we confirmed that monocytes isolated 4 h after in vivo endotoxin administration were irresponsive to ex vivo LPS stimulation (Fig. 1A). This effect was restored 7 days following endotoxemia. In contrast, monocyte cytokine responses to ex vivo LPS stimulation were unaffected in placebo subjects.

After in vivo endotoxin‐induced immunotolerance, monocytes showed a significant attenuated capacity to induce an oxidative burst response to *E. coli* and *S. aureus* (Fig. 1B). A similar trend toward *C. albicans‐*induced ROS release was observed. Finally, the microbial killing was investigated during endotoxin‐induced immunotolerance. After in vivo endotoxin challenge, monocytes showed a reduced *Candida* killing capacity in 2/5 subjects (Fig. 1C). The effect of endotoxin tolerance on microbial killing in the other volunteers was inconclusive, with one volunteer showing even enhanced monocyte‐mediated *Candida* killing during tolerance.

### 3.2 Endotoxin‐induced immunotolerance reduces the metabolic plasticity of monocytes

**Figure 2:**
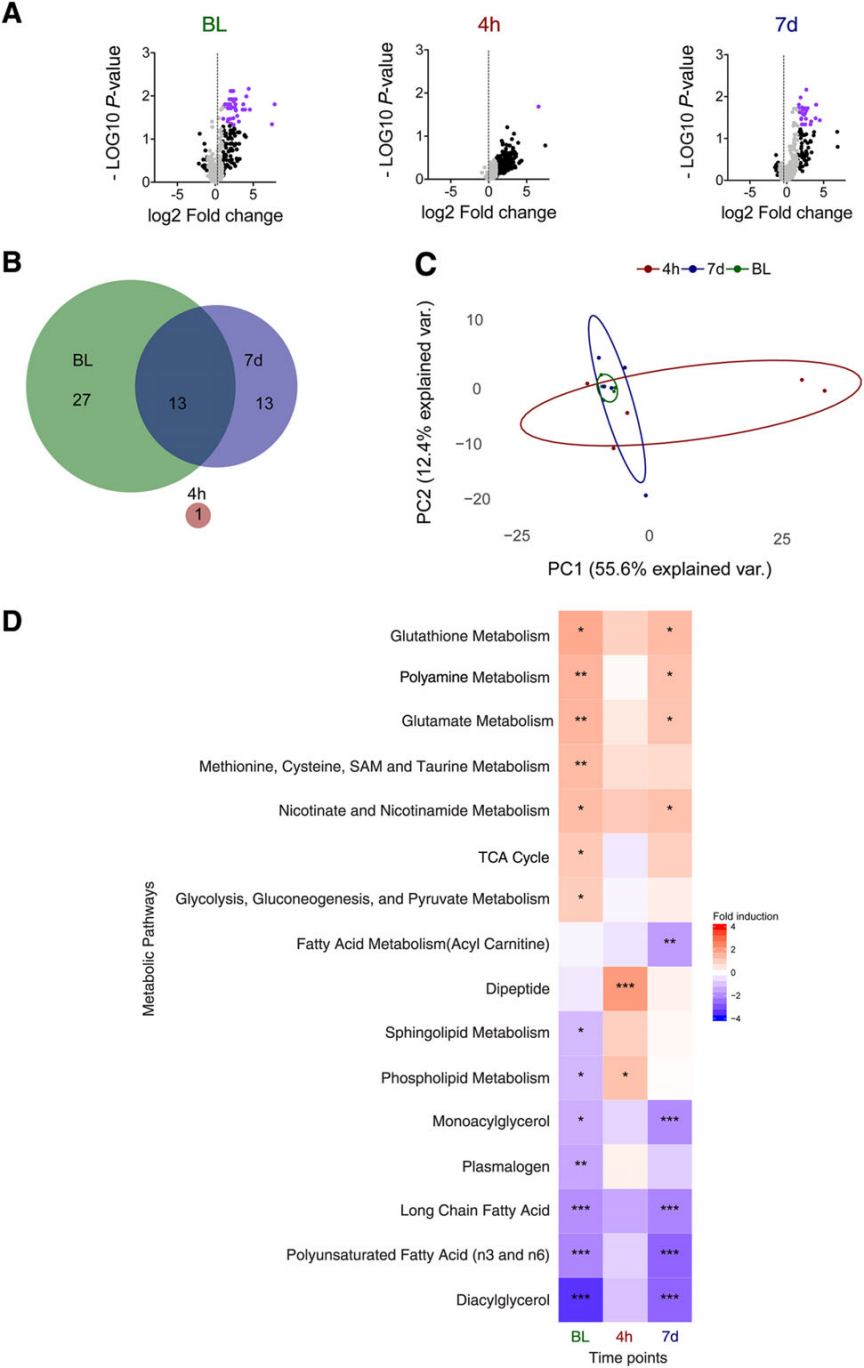
**Cellular metabolism during monocyte hyporesponsiveness**. (**A**) Volcano plots representing the –10log of the corrected *P*‐value (false discovery rate) and the relative mean log 2 fold change for cellular metabolites in monocytes from 5 human endotoxemia subjects. Each of the panels represents the comparison of ex vivo unstimulated versus ex vivo LPS‐stimulated CD14+ monocytes that were isolated at baseline (BL), 4 h, and 7 days following endotoxemia. Metabolites that demonstrated a mean log2 fold change > 1 or < –1 have been marked in black, and metabolites that additionally demonstrate a false discovery rate < 0.05 are marked in purple. (**B**) Venn diagram showing differentially regulated metabolites for all time points and their relevant overlap. (**C**) PCA analysis of the metabolic response to ex vivo LPS stimulation. In 5 endotoxemia subjects log2 fold changes of ex vivo LPS stimulated CD14+ monocytes were compared to unstimulated CD14+ monocytes isolated and analyzed for each time point of cell isolation. (**D**) Heat map of metabolite pathways that were differentially regulated by ex vivo LPS stimulation compared to ex vivo unstimulated CD14+ monocytes, following in vivo endotoxemia. The monocytes were isolated at different time points from 5 subjects before (BL), and 4 h (4h), or 7 days (7d) after intravenous administration of endotoxin. The mean log2 fold change of each of the groups is plotted for the various time points. For each pathway, different upregulated (red) or down‐regulated (blue) groups of metabolites are presented. *P < 0.05, **P < 0.01, and ***P < 0.001

Monocytes isolated from 5 volunteers that were infused with endotoxin were ex vivo LPS‐restimulated or left unstimulated for 24 h. Metabolic profiling of the monocytes was performed after stimulation at baseline, 4 h postendotoxin infusion, and 7 days postendotoxin infusion to characterize the metabolic responses to the ex vivo LPS stimulation. At baseline, when monocytes were still naive, ex vivo LPS stimulation resulted in a significant upregulation of 40 metabolites in monocytes (Fig. 2A and Table S1). In contrast, in immunotolerant monocytes obtained 4 h following in vivo endotoxemia, almost the full metabolic profile was down‐regulated compared to baseline in untreated monocytes (Fig. S1A, left panel). Also ex vivo LPS stimulation did not significantly upregulate abundance of metabolites; except for quinolinate, a downstream product of the kynurenine pathway, which was significantly increased (Fig. 2A, Table S1, and Fig. S1). Monocytes isolated 7 days following in vivo endotoxin administration showed only a partially recovered metabolic response upon ex vivo LPS stimulation. A total of 26 metabolites were significantly upregulated, of which 13 overlapped with the metabolic response at baseline (Fig. 2B and Table S1). Principal component analysis (PCA) demonstrated a high similarity of the metabolic response to ex vivo LPS stimulation at baseline, but a strong separation of the interindividual metabolic response during tolerance (Fig. 2C). At 7 days following in vivo endotoxemia, ex vivo LPS stimulation still did not demonstrated a homogeneous metabolic response between the subjects (Fig. 2C). Pathway analysis showed induction of multiple central metabolic pathways upon ex vivo LPS stimulation including glycolysis, pentose phosphate pathway (PPP), pyruvate metabolism, TCA cycle, glutamate metabolism, glutathione metabolism, and other circuits involved in amino acid metabolism (Fig. 2D). Furthermore, numerous pathways were down‐regulated, especially those involved in lipid metabolism such as biosynthesis of membrane‐bound lipids (phospholipids, sphingolipids, and glycerol‐based phospholipids) and fatty acid regulation (Fig. 2D). In contrast, endotoxin‐induced immunotolerance did not display modulation of these metabolic pathways after ex vivo LPS stimulation. Instead, the synthesis of dipeptides was highly induced (Fig. 2D). Metabolic plasticity of ex vivo LPS‐stimulated monocytes was only partially restored 7 days following in vivo endotoxemia with still key metabolic pathways dysregulated such as glycolysis, TCA cycle, methionine, cysteine, S‐adenosyl‐methionine and taurine metabolism, phospholipid metabolism, and sphingolipid metabolism. At this time point, ex vivo LPS stimulation induced down‐regulation of the fatty acid metabolism (acylcarnitine), whereas this pathway was not significantly affected at other time points (Fig. 2D), which highlights the changed metabolic response 7 days after endotoxin infusion.

### 3.3 Modulation of cellular metabolism in naive immune cells differentially influences cytokine responses

**Figure 3:**
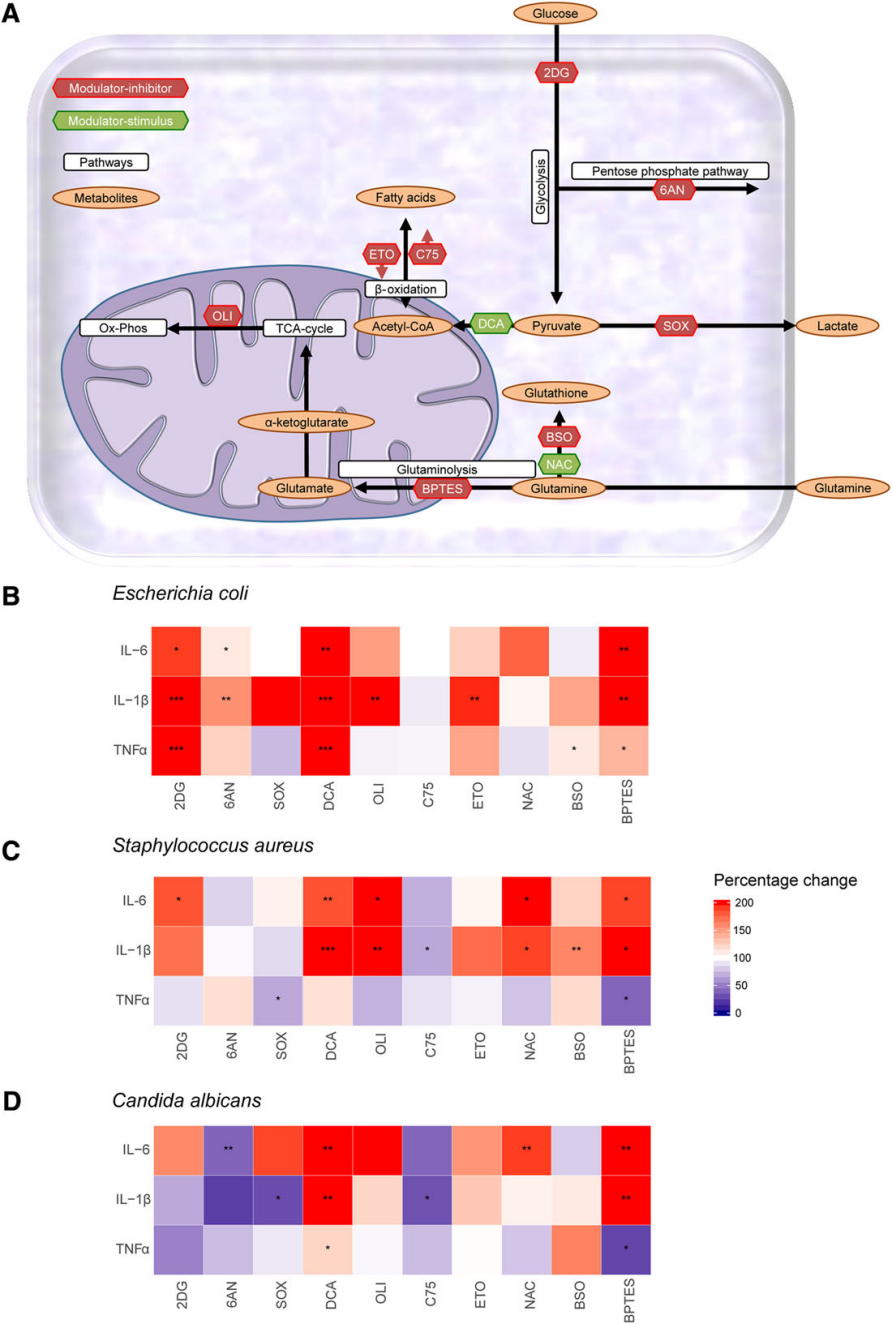
**Modulation of cellular metabolism influences monocyte cytokine responses**. (**A**) Schematic overview of the metabolic pathways that were targeted to study the effects of cellular metabolism on cytokine responses to microbial stimulation. Inhibitors of metabolic pathways (2DG, 6AN, SOX, OLI, C75, ETO, BSO, BPTES) are indicated with red labels and stimuli of metabolic pathways (DCA and NAC) with green labels. (**B–D**) Percentage change in TNF‐α, IL‐1β, and IL‐6 responses for *E. coli* (**B**), *S. aureus* (**C**), and *C. albicans* (**D**) following inhibition (2DG, 6AN, SOX, OLI, C75, ETO, BSO, BPTES) or stimulation (DCA and NAC) of designated metabolic pathways compared to the vehicle control of the inhibitor. 2DG (glycolysis, *n *= 19), 6AN (PPP, *n *= 11), SOX (pyruvate metabolism, *n* = 15), DCA (TCA cycle, *n *= 20), OLI (ATP synthesis, *n *= 10), C75 (fatty acid metabolism, *n *= 13), ETO (fatty acid metabolism, *n *= 12), NAC (glutathione synthesis, *n *= 19), BSO (glutathione synthesis, *n *= 12), and BPTES (glutaminolysis, *n *= 12). Data are shown as the median with *P*‐values of statistical comparison by the Wilcoxon signed rank test (*P < 0.05, **P < 0.01, and ***P < 0.001)

The metabolic profiling of monocytes indicated a significant loss of metabolic plasticity in immunotolerant monocytes. Therefore, the influence of cellular metabolism on immune functions including cytokine production, oxidative burst, microbial (*Candida*) killing, and cell viability were determined. Metabolic pathways affected by immunotolerance were systematically targeted by inhibitors/modulators at crucial steps in the metabolic pathways (Fig. 3A and Table 1). The effect of each metabolic modulation on cell viability was measured after 24 h and did not reveal reduced cell viability compared to their respective vehicle control (Fig. S2). In endotoxin‐induced tolerant monocytes, loss of metabolic plasticity correlated with impaired ex vivo cytokine responses (Figs. 1A and 2D). However, proinflammatory cytokine responses induced by *E. coli*, *S. aureus*, and *C. albicans* showed different results after modulation of individual metabolic pathways in vitro (Fig. 3B–D). None of the metabolic inhibitions reduced *E. coli*‐induced cytokine responses as expected, but instead increased cytokine responses were observed (Fig. 3B). Inhibition of glutaminolysis with bis‐2‐(5‐phenylacetamido‐1,3,4‐thiadiazol‐2‐yl)ethyl sulfide (BPTES) significantly reduced TNF‐α concentrations in response to *S. aureus* and *C. albicans* (Fig. 3C and D). Inhibition of the PPP with 6‐aminonicotinamide (6AN) significantly reduced *C. albicans‐*induced IL‐6 production. Inhibition of glycolysis with 2‐deoxy‐d‐glucose (2DG) showed a trend in reduced *C. albicans*‐induced TNF‐α and IL‐1β production (Fig. 3D), but increased *E. coli*‐induced cytokine responses. Notable, cytokine responses were consistently induced for all three microbial stimuli after stimulation of the TCA cycle with potassium dichloroacetate (DCA) (Fig. 3B–D).

### 3.4 Glycolysis, PPP, and glutaminolysis are crucial for oxidative burst

**Figure 4:**
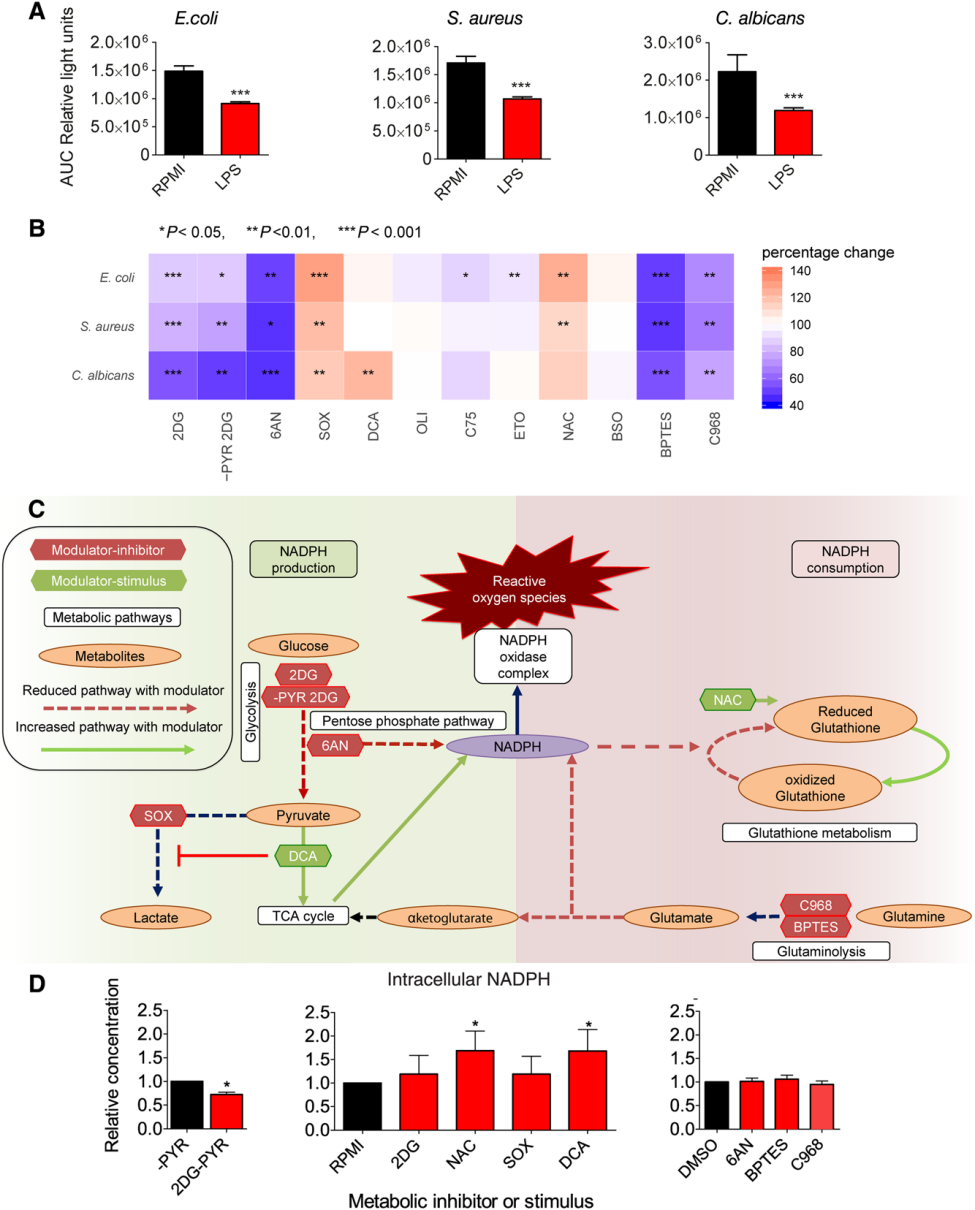
**Impact of modulation of metabolic pathways on the production of ROS**. (**A**) In vitro ROS production in PBMCs after 24 h of preincubation with LPS (red bars) compared to vehicle control (black bars) upon stimulation with *E. coli, S. aureus*, and *C. albicans* (*n *= 20). Data are expressed as AUC of emitted light after oxidation of luminescence, presented in mean relative light units with a statistical comparison by the Wilcoxon signed rank test (***P < 0.001). (**B**) Percentage change in ROS production following inhibition (2DG, ‐PYR 2DG, 6AN, SOX, OLI, C75, ETO, BSO, BPTES, and C968) or stimulation (DCA or NAC; green labels) of designated metabolic pathways compared to the vehicle control of the modulator. 2DG (glycolysis, *n *= 19), ‐PYR 2DG (glycolysis, *n *= 8), 6AN (PPP, *n *= 13), SOX (pyruvate metabolism, *n *= 14), DCA (TCA cycle, *n *= 18), OLI (ATP synthesis, *n *= 10), C75 (fatty acid metabolism, *n *= 9), ETO (fatty acid metabolism, *n *= 15), NAC (glutathione synthesis, *n *= 14), BSO (glutathione synthesis, *n *= 12), BPTES (glutaminolysis, *n *= 19), and C968 (glutaminolysis, *n *= 7). Data are shown as the median with *P*‐value of statistical comparison by the Wilcoxon Signed Rank Test (*P < 0.05, **P < 0.01, and ***P < 0.001). (**C**) Simplified schematic overview of the metabolic pathways that were targeted with inhibitor (green labels) or stimulator (red labels) that could lead to increased intracellular NAPDH levels (green arrows) or decreased (red dotted line) NADPH levels. (**D**) Relative changes in monocyte intracellular NADPH levels measured by colorimetric assay following treatment of the cells for 24 h with various modulators of cellular metabolism (*n *= 6, red bars). Fold changes are relative to the appropriate vehicle control (black bars; either RPMI, or RPMI without pyruvate [‐PYR], or DMSO). Bars represent the mean ± sem and were compared for significance using the Wilcoxon Signed Rank Test (*P < 0.05)

The oxidative burst of in vitro LPS‐tolerized PBMCs was assessed to confirm that immunotolerance corresponds with reduced oxidative responses. Indeed reduced ROS release upon microbial restimulation was observed in LPS‐tolerized PBMCs (Fig. 4A). The oxidative burst was assessed by measuring ROS generation in response to *E. coli, S. aureus*, and *C. albicans* after inhibition or simulation of various metabolic pathways in monocytes. ROS release was diminished upon inhibition of glycolysis with 2DG, PPP with 6AN, and glutaminolysis with BPTES or C968. In contrast, inhibition of lactate production with sodium oxamate (SOX) augmented ROS release. Increasing TCA‐cycle activity with DCA increased oxidative burst in response to *C. albicans* exclusively. Albeit being an antioxidant, *N*‐acetylcysteine (NAC) also increased oxidative burst in response to microbial stimulation (Fig. 4B). Inhibition of pathways known to fuel NADPH (glycolysis, PPP, and glutaminolysis) reduced ROS release, while modulation of pathways leading to increased NADPH levels (DCA and NAC) increased ROS release (Fig. 4C). Measurements of intracellular NADPH levels following modulation of these NADPH‐dependent pathways validated this. The blockade of glycolysis using 2DG reduced intracellular NADPH levels if pyruvate was absent in the media, whereas NAC and DCA increased intracellular NADPH levels (Fig. 4D). No changes in intracellular NADPH concentrations were detected by the inhibition of PPP or glutaminolysis.

### 3.5 Immunotolerance is associated with reduced *Candida* killing capacity

**Figure 5:**
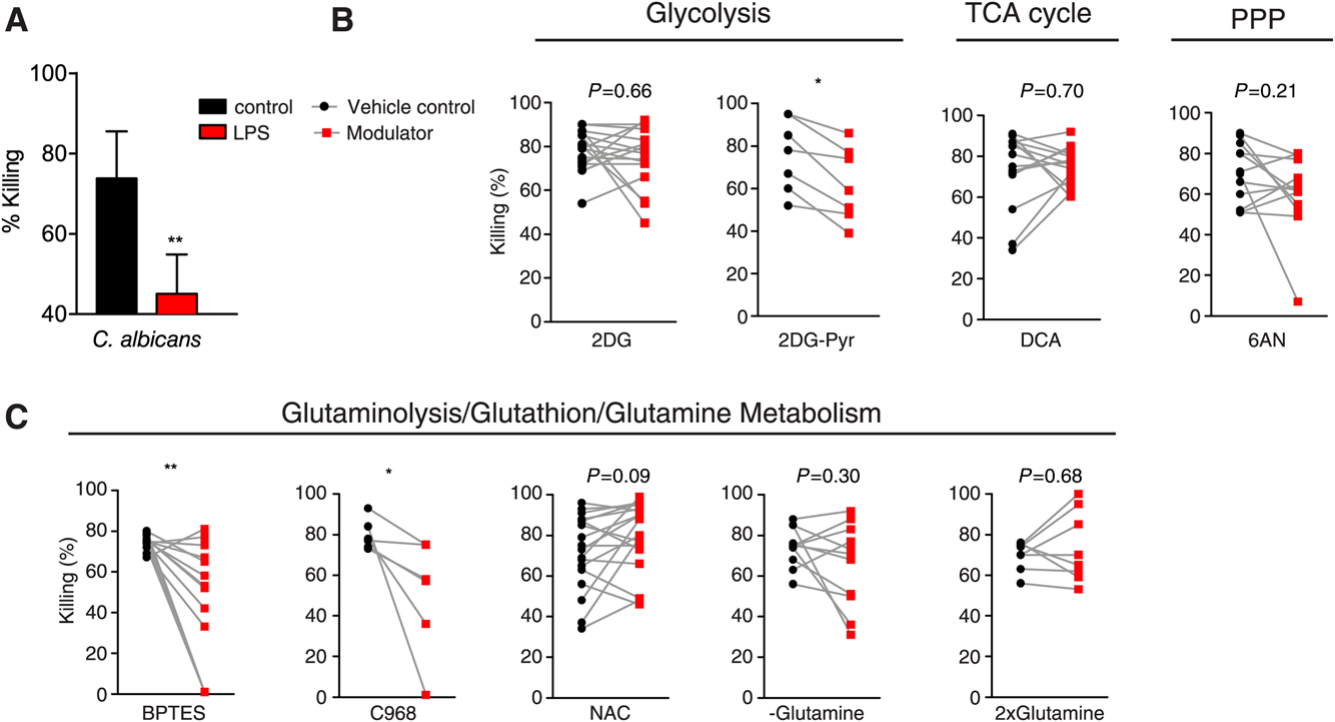
**Intracellular microbial killing in vitro**. (**A**) In vitro microbial killing capacity by PBMCs after incubation with *C. albicans* (*n *= 8) with 24 h pre‐exposure (red bars) to LPS compared to media control without LPS (black bars). (**B,C**) In vitro *C. albicans* killing in PBMCs after 24 h pre‐exposure to modulators (red bars) of (**B**) glycolysis (2DG, *n *= 16, 2DG‐PYR, *n *= 7) TCA cycle (DCA, *n* = 14), PPP (6AN, *n = *12), (**C**) glutaminolysis (BPTES, *n *= 16 or C968, *n = *6), glutathione synthesis (NAC, *n* = 19), or glutamine metabolism (‐glutamine, *n *= 11, 2xglutamine, *n *= 8) compared to the unmodulated situation. All data in this figure are presented as the mean ± sem and with a significant *P*‐value of statistical comparison by the Wilcoxon signed rank test (*P < 0.05, **P < 0.01)

In vitro experiments showed a significantly reduced killing capacity for *C. albicans* in LPS‐induced tolerant monocytes (Fig. 5A). Inhibition of glycolysis (2DG) did consistently reduce *Candida* killing capacity in healthy donors, but only when pyruvate was excluded from the culture media during the preincubation period (Fig. 5B). Modulation of the TCA cycle and PPP did influence *Candida* killing but showed high variability between donors. We found that inhibition of glutaminolysis (BPTES or C968) exhibited a substantial impact on *Candida* killing in naive monocytes (Fig. 5C). Glutathione precursor NAC augmented killing capacity in the majority of the donors, whereas other donors showed unchanged killing capacity. In some donors, killing capacity was even reduced upon NAC treatment. Remarkably, experiments with reduced or increased glutamine levels in culture media demonstrated similar trends.

### 3.6 Reduced oxidative burst in monocytes correlates with defective microbial killing mechanisms

**Figure 6:**
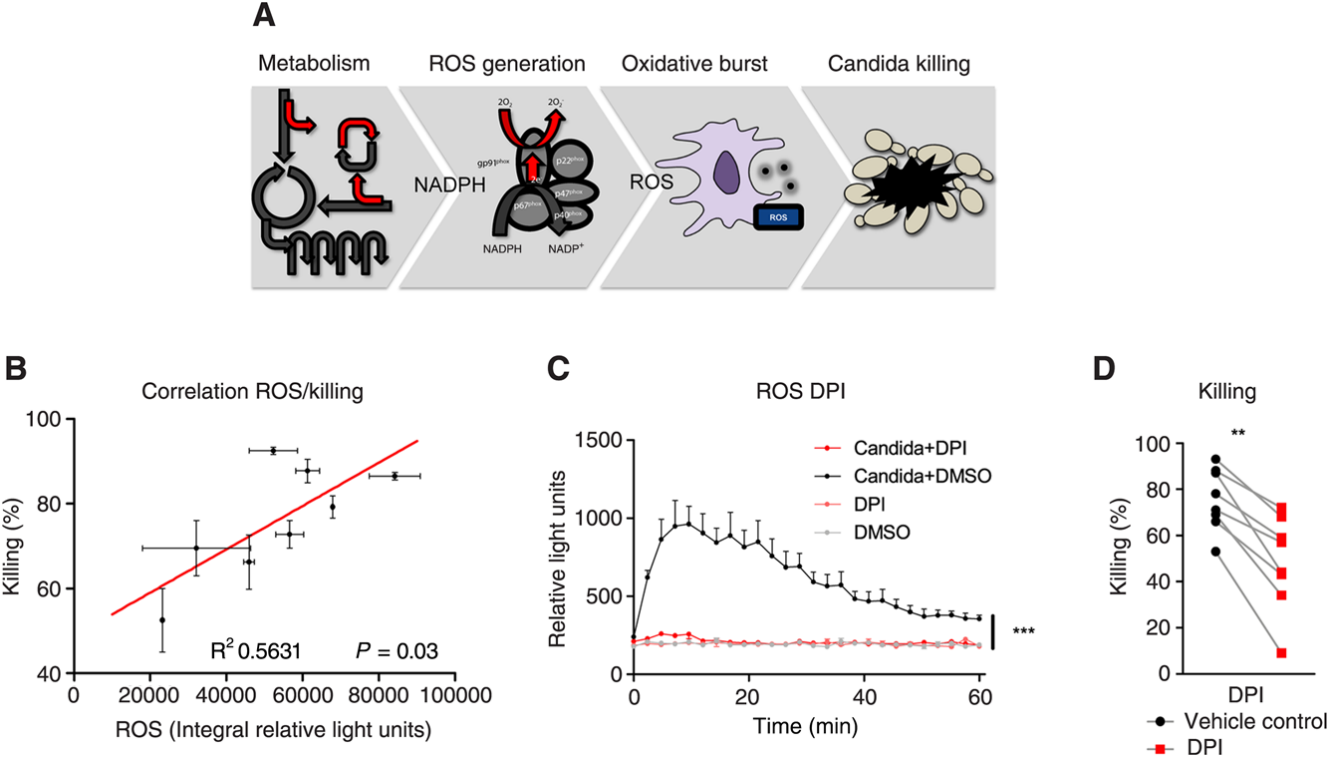
**Capacity to mount oxidative responses correlates to microbial killing**. (**A**) Schematic presentation of the hypothesis how metabolic changes can influence the production of ROS (oxidative burst) and monocyte *C. albicans* killing capacity. (**B**) Correlation of oxidative burst assessed as the integral of the luminesce signal following luminol conversion in response to *C. albicans* stimulation and *C. albicans* killing capacity in the percentage of killed fungal CFUs in PBMCs of healthy donors (*n = 6*). (**C**) PBMCs of healthy donors preincubated with vehicle control (DMSO black/gray lines) or DPI (red lines) for 24 h and subsequently stimulated with opsonized live *C. albicans* or left unstimulated. Oxidative burst generation was expressed as luminescence conversion over measured over time (measured every 145 s, during 60 min). (**D**) *C. albicans* killing capacity in PBMCs of the same 6 donors after 24 h pre‐exposure to DPI, compared to the unmodulated situation (vehicle control). All data in this figure are presented as the mean ± sem and with a significant *P*‐value of statistical comparison by the Wilcoxon signed rank test (*P < 0.05, **P < 0.01)

Our data demonstrate that central metabolic pathways like glutaminolysis and glycolysis are both involved in ROS generation (Fig. 4B) and candida killing in naïve monocytes (Fig. 5B and C). The importance of ROS production for host defense is demonstrated in patients, with genetic defects in the NADPH oxidase complex, who are unable to produce ROS and are highly susceptible to staphylococcal and *Aspergillus* infections.^24^ Hypothetically, metabolic modulation might have influenced monocyte antimicrobial functions in similar ways; by altering NADPH‐dependent oxidative burst, thereby influencing phagocytic activity and killing capacity (Fig. 6A). To test this hypothesis, we investigated the correlation between *C. albicans*‐induced oxidative burst and fungal killing (Fig. 6B). In these experiments, *C. albicans* killing was measured in the presence of diphenyleneiodonium (DPI). DPI is a frequently used and potent ROS inhibitor mediated by flavoenzymes, in particular NADPH oxidase.^25^ As expected, DPI‐treated monocytes demonstrated drastically reduced capacity to mount an oxidative burst (Fig. 6C). In parallel, we observed a reduced capacity of monocytes to kill *C. albicans* yeasts, confirming the link between ROS and killing (Fig. 6D).

## 4 DISCUSSION

This study revealed impaired plasticity of monocytes to modify their metabolism in response to immunogenic stimulation following in vivo endotoxin‐induced immunotolerance. This was accompanied by reduced cytokine responses and ROS release, which in some individuals was associated with impaired microbial (*C. albicans*) killing. During endotoxin‐induced immunotolerance, most major metabolic pathways failed to be modulated in monocytes upon ex vivo LPS restimulation. Even 7 days after endotoxin‐induced immunotolerance, major metabolic pathways including glycolysis and the TCA cycle were not inducible. This implies a sustained defect in metabolic reprogramming following in vivo exposure to a bolus endotoxin infusion. Validation in naive immune cells showed that these pathways were essential for the immune response to microbial ligands, highlighting the influence of metabolic modulation on essential antimicrobial functions in immunotolerance.

Dysregulated glycolysis and oxidative phosphorylation was postulated as an explanation for the immunological defects of tolerant monocytes.^14^ Recently, the metabolic‐rewiring signatures during immunotolerance have been identified in LPS‐treated THP‐1 cells.^15^ In line with these findings, we showed a homogeneous metabolic response of naïve monocytes to ex vivo LPS stimulation. Similarly, we observed significant differences in metabolic adaptation between unstimulated and LPS‐stimulated monocytes in the progression of activation to tolerant and resolution of the inflammatory state. In contrast to TPH‐1 cells,^15^ we observed heterogeneity in the metabolic responsiveness during immunotolerance in healthy subjects. This suggests that metabolic remodeling is individual‐specific. These changes may even differently correlate with alterations in antimicrobial immune functions on an individual level. Therefore, individual‐specific metabolic reprogramming might have contributed to the interindividual variation in monocyte‐mediated *C. albicans* killing among volunteers infused with endotoxin. When studying sepsis patients, the interindividual variability may be even more considerable, as a result of differences in underlying disease and sepsis severity. Therefore, we propose the need for large multicenter studies that correlate the interindividual variability of metabolic reprogramming with the capacity of monocytes to kill microbes. Such multicenter clinical trials may have enough power to identify metabolic biomarkers that help to identify high‐risk individuals with a predisposition to secondary infections.

In an attempt to translate pathophysiological mechanisms into clinical practice, we used an in vivo endotoxemia model to simulate the biphasic response of sepsis. This model is widely used to study the early and transient phase of sepsis, illustrated by a controlled systemic inflammatory response in healthy subjects.^26^ Endotoxin challenge induces an immunotolerant state where immune cells are refractory to ex vivo stimulation, which bears hallmarks of the anti‐inflammatory response seen in sepsis.^11^ This model enabled the evaluation of comprehensive and complex metabolic rewiring in vivo during the biphasic inflammatory response as present in sepsis. However, there are noteworthy differences between experimental endotoxemia and clinical sepsis that underlines the caution that should be taken with extrapolation of the data to clinical sepsis.

Systemic endotoxin‐induced responses in this model are milder than that suffered by septic patients, who by definition suffer from organ injury. This is reflected by the fact that endotoxin‐challenged volunteers recover fully without residual deficits and can be discharged 8 h after challenge.^16^, ^18^, ^19^ In contrast, sepsis‐associated organ dysfunction is often life‐threatening and assessed in all patients suspected of sepsis.^27^ Moreover, severe sepsis survivors have an increased risk of long‐term deficits including physical and cognitive impairment.^28^ Given that, in experimental endotoxemia, already major (long‐lasting) defects in metabolic plasticity of monocytes were observed, we envision that during sepsis the severe impact on the immune system may trigger even more severe changes in cellular metabolism. This possibly contributes to the sustained immune dysfunction in septic patients, who develop secondary infections days to weeks after diagnosis.^7^, ^29^

Of note, systemic LPS stimulation can result in opposite immune responses in vivo.^30^ Several studies showed that LPS priming with low concentrations could augment immune responses, resembling in a proinflammatory phenotype.^30^, ^31^, ^32^ However, in line with other endotoxemia studies, we showed a proper immunotolerant phenotype in the current study.^16^, ^18^, ^19^

Essential metabolic differences were found between homeostasis and endotoxin‐induced immunotolerance, which may reveal biomarkers or therapeutic targets for immunoparalysis. The untargeted metabolome analysis identified the metabolites quinolinate and kynurenine to be upregulated, respectively 4 h and 7 days after endotoxin administration. These are metabolites of the tryptophan‐kynurenine pathway, which is known to be activated or dysregulated in several inflammatory conditions including infectious diseases, autoimmune disorders, malignancy, and cardiovascular diseases.^33^ Accumulation of kynurenine and downstream metabolites, through increased tryptophan degradation by IDO‐1, contributes to hypotension due to vasodilatation, and is therefore considered as a novel target for septic shock treatment.^34^ Besides these vascular effects, alterations in this pathway correlate with disease severity and impaired immune function.^35^ Although kynurenines might be linked to immunoparalysis, whether these metabolites individually can function as a target to improve immunological function in immunotolerant cells is yet to be explored.

Cytokine responses are crucial for activation of immune responses, immune cell recruitment, and initiation of antimicrobial functions. We confirmed attenuated ex vivo cytokine responses during immunotolerance. Nevertheless, inhibition of specific metabolic pathways, identified by induction of immunotolerance, did not uniformly impair cytokine responses in naive monocytes. Several studies have examined the effects of glucose metabolism upon LPS or bacterial stimulation and showed diverse cellular reprogramming depending on the stimulus, experimental setup, and cell population.^14^, ^36^, ^37^, ^38^, ^39^ Glycolysis is essential for host defense against *C. albicans* infection.^40^ Glycolysis inhibition decreased cytokine response to *C. albicans* stimulation, but increased upon *E. coli* or *S. aureus* stimulation in our experiments. These divergent cytokine responses may be the result of differences in pathogen recognition receptor‐mediated signaling pathways, as well as metabolic changes, induced upon stimulation with crucially different microbes.^39^

Oxidative responses are classically linked to the early hyperinflammatory phase of sepsis and associate with increased organ damage.^41^ However, during monocyte immunotolerance, impaired ROS production was observed,^42^ and attenuated superoxide production was reported after ex vivo restimulation of monocytes from septic patients.^43^ In line with this, we observed impaired oxidative burst in immunotolerant monocytes, both ex vivo and in vitro. The metabolic routes involved in ROS production are glycolysis, PPP, glutaminolysis, TCA cycle, and glutathione synthesis. Modulation of NADPH‐yielding pathways, including glycolysis, glutathione metabolism, or the TCA cycle influenced intracellular NADPH levels in our experiments significantly. However, the PPP and glutaminolysis are considered essential sources of NADPH generation for cells^44^, ^45^, ^46^; controversially, these were not significantly altered in our experiments upon inhibition.

Oxidative burst is crucial for antimicrobial defense.^24^ In line with this, we observed a correlation between killing capacity and oxidative burst in naive monocytes. Similarly, modulation of glutaminolysis and glycolysis modified both oxidative burst as well as the monocyte killing capacity. These results support the notion that metabolism can directly influence immune function, by influencing oxidative burst and microbial (*Candida)* killing capacity. At the same time, metabolic modulation induced high variability in intracellular killing between donors. This can partly be explained by the experimental design, in which inhibitors are washed away after the preincubation period to prevent interference with the metabolic pathways of *C. albicans*. This probably enabled complete or partial restoration of indirectly targeted metabolic pathways, like glycolysis (2DG) or glutamine metabolism, as these compounds can compete with nutrients in the culture media like glucose, pyruvate, and glutamine. In contrast, direct targeting of enzymes like GLS‐1 inhibition by BPTES or C968 may induce less transient metabolic reprogramming.

Glutamine has gained clinical interest as it has regulatory effects on immune cell function and may serve as a beneficial supplement in critically ill patients.^47^ We observed variation in monocyte killing capacity in glutamine poor or supplemented conditions. In line with this, trials in critically ill patients similarly observed divergent outcomes upon glutamine supplementation.^48^, ^49^ Some studies demonstrated benefits in critically ill patients, including decreased mortality and infection risk,^50^, ^51^, ^52^ but it is now debated that glutamine supplementation can further elucidate hyperinflammation.^53^ The benefit of modulating glutamine metabolism, therefore, may depend on the individual inflammatory state. As interference of cellular metabolism can have unpredictable outcomes, studies focusing on immune cells metabolism and the relation to immune function are warranted, to avoid harm and identify the patients that could benefit from targeted therapy.

Remarkably, DCA (TCA cycle stimulator) augmented cytokine responses to all microbial stimuli in our study. Similar effects were observed after modulation with NAC (glutathione synthesis). After exposure to NAC, monocytes also demonstrated augmented oxidative burst upon *S. aureus* or *E. coli* stimulation. This was similar for DCA upon *C. albicans* stimulation. These beneficial effects support the potential of these modulators as novel candidates to reverse immunoparalysis and will be discussed further in this context.

First, NAC is commonly known as a ROS scavenger and appreciated for its capacity to reduce oxidative stress and organ damage as well as anti‐inflammatory effects. NAC was introduced as an antioxidant in SIRS (systemic inflammatory response syndrome) and sepsis cohorts, but was ineffective in reducing mortality.^54^ Conversely, high NAC levels can increase ROS generation in endothelial cells,^55^ by fueling glutathione synthesis, this probably results in a pro‐oxidant shift in the glutathione redox state. This pro‐oxidant effect is in line with our results where in most donors augmented antimicrobial functions were observed with relatively high NAC concentrations.

Second, DCA was used in a clinical trial to treat hyperlactatemia, which included patients with septic shock, but did not improve clinical outcome.^56^ Besides reducing lactate levels, DCA activates the pyruvate dehydrogenase complex (PDC) and stimulates mitochondrial glucose oxidation through increased mitochondrial pyruvate influx.^57^ DCA in a sepsis mouse model promoted restoration of immunometabolism, increased survival, and improved bacterial clearance.^58^ New insights and the data presented here suggest that the modulators DCA and NAC might be reconsiderable targets in sepsis treatment. However, the aforementioned trials with DCA and NAC focused on the hyperinflammatory aspects of sepsis. One can speculate that timing of treatment and the heterogeneity of populations enrolled in these trials have concealed beneficial effects in patients subgroups, in particular for patients with profound immunosuppression. This provides a rationale for future studies on the use of these metabolic modulators in patients with sepsis‐induced immunoparalysis.

Our study has some limitations. First, metabolomic profiling was performed in a small number of participants. Therefore, validation experiments were focused on metabolic pathways rather than on single metabolites. We assume that parallel to pathway analyses, individual metabolites may help to identify novel metabolic therapeutic targets. Second, our study population was within an age range of 18–35, whereas 64.9% of cases of sepsis occurs in patients at or over the age of 65.^59^ Immune metabolism is a research field that is relatively new, and knowledge on the differences in immunometabolic reprogramming between adults and the elderly is scarce. Our results may, therefore, not be generalizable to populations that commonly develop sepsis. Third, our sepsis model focused on the activation of the innate immune system, although sepsis‐induced immunoparalysis is characterized by defects in both innate and adaptive immunity. Fourth, our in vitro model was limited to modulate single metabolic pathways separately. Modulation may also have led to compensatory or alternative mechanisms for metabolic adaptation. Therefore, this study does not provide information about the collateral interactions of various metabolic processes or their connections with molecular, epigenetic, and transcriptional changes, which all contribute to immunotolerance. Considering that metabolic modulation might provide new therapeutic options for sepsis, a systematic biological approach with validation in larger endotoxemia and patient cohorts is necessary to unravel the complexity of metabolic reprogramming and confirm the therapeutic value of metabolic targets in immune paralysis.

To conclude, this study provides essential insights into metabolic changes in immunotolerant monocytes, and we report a loss of metabolic plasticity in these cells. By modulation of metabolic pathways in naive immune cells, we validated the concept that metabolic pathways changed in immunotolerance can affect antimicrobial functions. Furthermore, several metabolites and metabolic pathways were identified that might represent future therapeutic targets to reverse sepsis‐induced immunoparalysis. As such, this study opens up new therapeutic avenues for sepsis‐induced immunoparalysis.

## DISCLOSURE

The authors declare no conflicts of interest.

## Supporting information

Figure S1: Changes in metabolites during endotoxin‐induced monocyte tolerance.
Figure S2: Effect of metabolic modulation on PBMC viability.

Table S1. Differentially regulated metabolites following ex vivo LPS stimulation.
