# Supplementary material for: Frontline Science: Endotoxin‐induced immunotolerance is associated with loss of monocyte metabolic plasticity and reduction of oxidative burst
Source: J Leukoc Biol. 2019 Jun 6;106(1):11–25. doi: 10.1002/JLB.5HI0119-018R (PMC6852552; doi:10.1002/JLB.5HI0119-018R)
Supplement: Supplementary file 1 — Figure S1: Changes in metabolites during endotoxin‐induced monocyte tolerance. Figure S2: Effect of metabolic modulation on PBMC viability. [file JLB-106-11-s002.docx]

**Supplemental Figures**

**
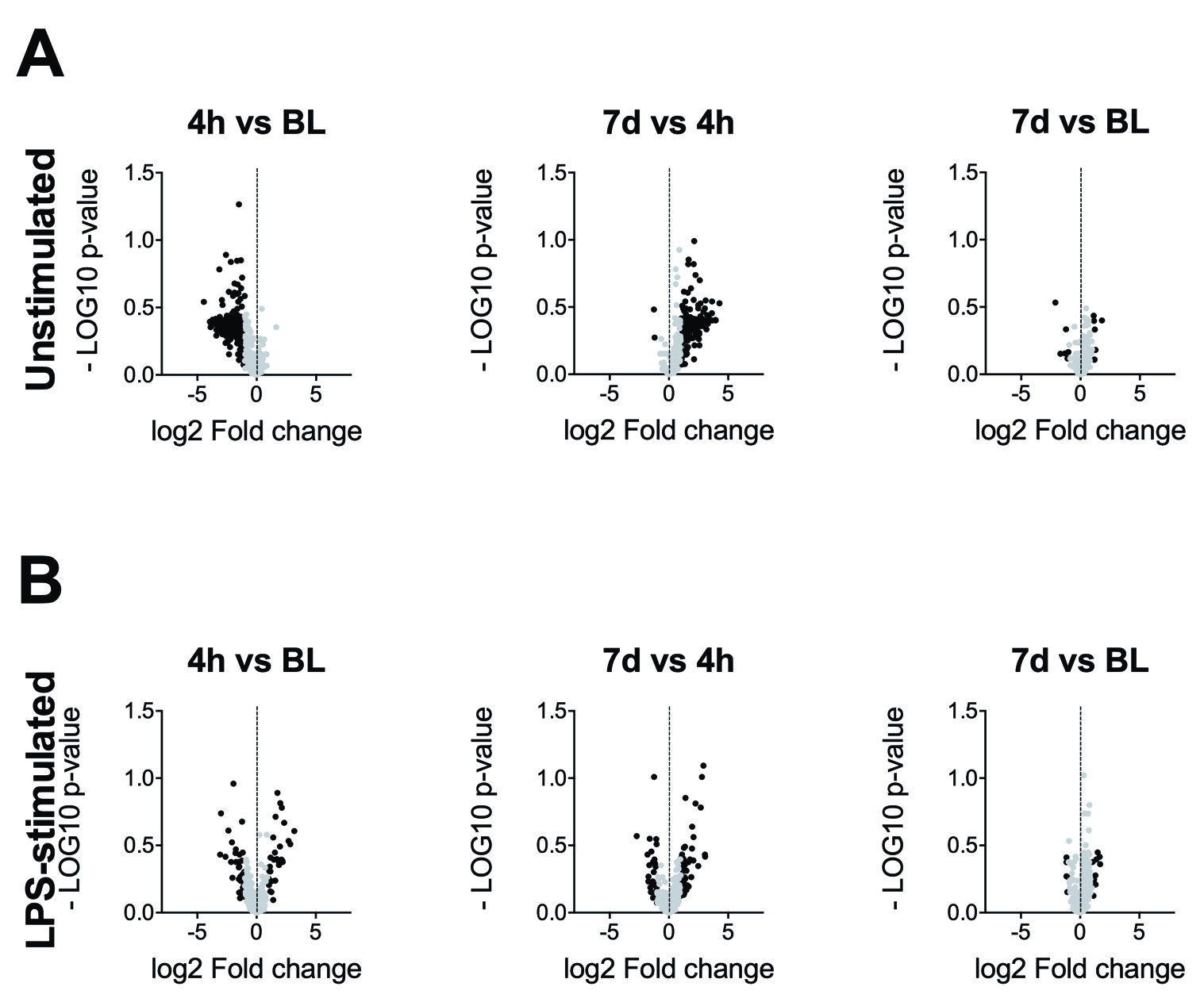
**

**Figure S1: Changes in metabolites during endotoxin-induced monocyte tolerance.**

(A, B) Volcano plots representing the -10log of the corrected p-value (false discovery rate) and the relative mean log 2 fold change for cellular metabolites in (A) unstimulated or (B) LPS stimulated CD14+ monocytes that were isolated at baseline, 4 hours, and 7 days following endotoxemia. Comparisons of (A) ex vivo unstimulated monocytes and (B) ex vivo LPS stimulated monocytes were made between the three timepoints baseline (BL), 4 hours (4h) and 7 days (7d). Metabolites that demonstrated a mean log2 fold change > 1 or < -1 have been marked in black.


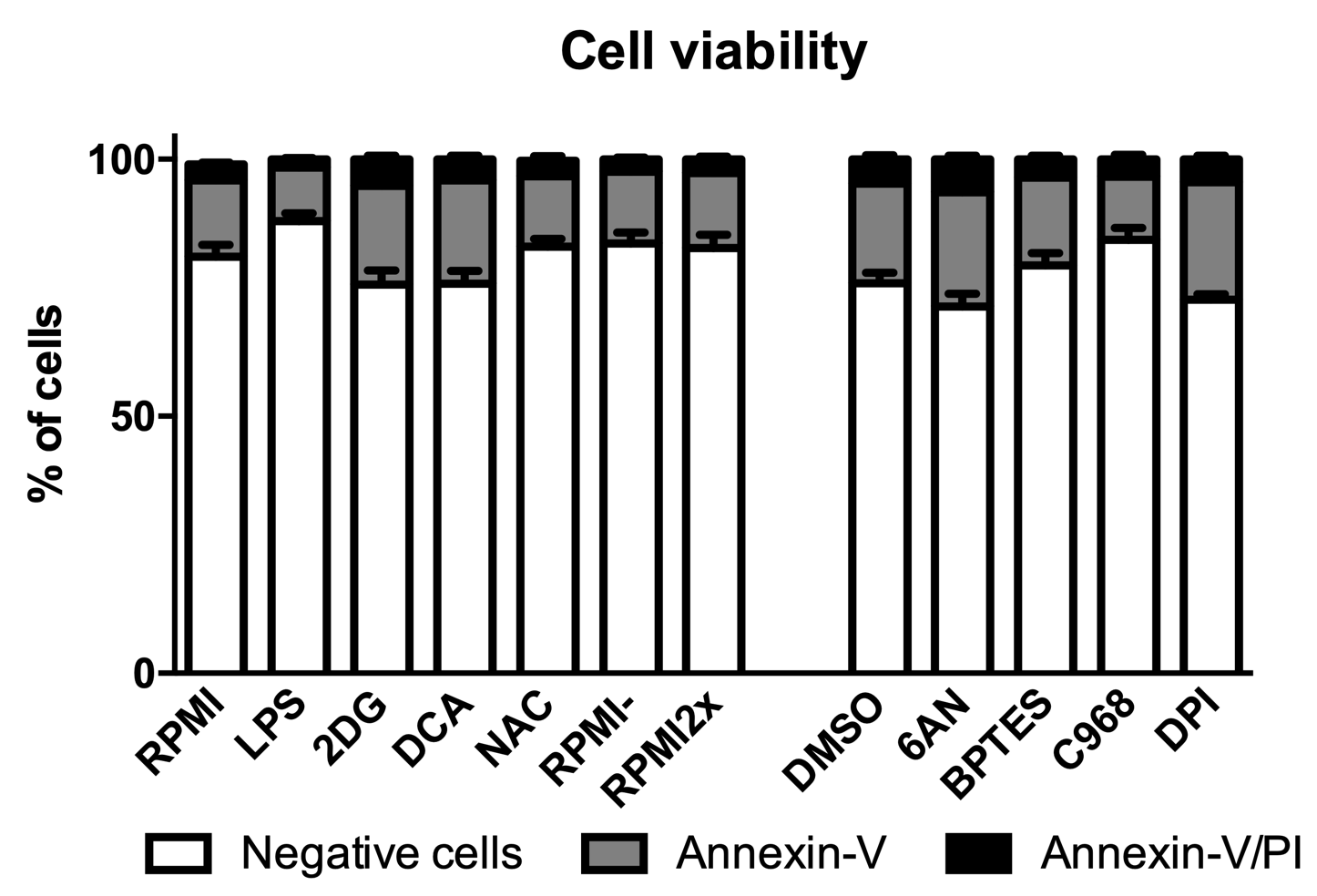


**Figure S2: Effect of metabolic modulation on PBMC viability.**

Viability of PBMCs assessed by Annexin-V and PI staining after 24 hours modulation of specific metabolic pathways and their respective vehicle controls. Live cells are depicted in white and are negative for Annexin V and PI. The percentage apoptotic cells are visualized by Annexin-V staining in grey. Late apoptotic cells, both positive for Annexin V and PI, are depicted in black.
