# Supplementary material for: Frontline Science: Endotoxin‐induced immunotolerance is associated with loss of monocyte metabolic plasticity and reduction of oxidative burst
Source: J Leukoc Biol. 2019 Jun 6;106(1):11–25. doi: 10.1002/JLB.5HI0119-018R (PMC6852552; doi:10.1002/JLB.5HI0119-018R)
Supplement: Supplementary file 2 — Table S1. Differentially regulated metabolites following ex vivo LPS stimulation. [file JLB-106-11-s001.docx]

**Table S1. Differentially regulated metabolites following ex vivo LPS stimulation.**

| **Baseline** | | |  | **4 hours** | | |  | **7 days** | | |
| --- | --- | --- | --- | --- | --- | --- | --- | --- | --- | --- |
| **Metabolite** | **Log 2 fc** | **P-value** |  | **Metabolite** | **Log 2 fc*_a_*** | **P-value** |  | **Metabolite** | **Log 2 fc** | **P-value** |
| 2-Hydroxyglutarate | 1.874026 | 0.01192 |  | Quinolinate | 6.575481 | 0.020694 |  | 15- Hydroxyeicosatetraenoic acid (15-HETE) | 2.357759 | 0.027306 |
| 2'-Deoxyinosine | 3.920577 | 0.020694 |  |  |  |  |  | 3-Hydroxy-3-methylglutarate | 1.608587 | 0.018845 |
| 3-(4-Hydroxyphenyl)lactate | 1.967203 | 0.015651 |  |  |  |  |  | 3-Sulfo-L-alanine | 2.074977 | 0.018168 |
| 3-Phosphoglycerate | 1.677196 | 0.015651 |  |  |  |  |  | 4-Guanidinobutanoate | 2.403702 | 0.019354 |
| 4-Guanidinobutanoate | 2.063582 | 0.019354 |  |  |  |  |  | Acetylphosphate | 2.648706 | 0.021693 |
| 7-Methylguanine | 1.665069 | 0.015651 |  |  |  |  |  | Cysteine-glutathione disulfide | 2.507803 | 0.046071 |
| Adenosine 5'-diphosphoribose (ADP-ribose) | 2.708333 | 0.020694 |  |  |  |  |  | Cysteinylglycine | 1.99448 | 0.033043 |
| Adenosine 5'-monophosphate (AMP) | 1.646716 | 0.039194 |  |  |  |  |  | Deoxycarnitine | 1.872394 | 0.010473 |
| Ascorbate (Vitamin C) | 2.891406 | 0.029004 |  |  |  |  |  | Glutathione, oxidized (GSSG) | 2.598001 | 0.033856 |
| Beta-guanidinopropanoate | 1.969989 | 0.033856 |  |  |  |  |  | Glycerol 3-phosphate | 1.649953 | 0.015651 |
| Carnitine | 2.370578 | 0.032855 |  |  |  |  |  | Glycerophosphoethanolamine | 1.744632 | 0.023571 |
| Cysteine-glutathione disulfide | 3.696881 | 0.015651 |  |  |  |  |  | Glycerophosphorylcholine (GPC) | 2.051144 | 0.046071 |
| Cytidine-5'-diphosphoethanolamine | 7.828593 | 0.015651 |  |  |  |  |  | Itaconate | 4.492276 | 0.036713 |
| Deoxycarnitine | 2.021456 | 0.007703 |  |  |  |  |  | Kynurenine | 3.922392 | 0.032855 |
| Dihydroxyacetone phosphate (DHAP) | 2.579124 | 0.018664 |  |  |  |  |  | N-Acetylcysteine | 3.979615 | 0.015651 |
| Erythronate | 1.691603 | 0.034764 |  |  |  |  |  | N-Acetylglutamate | 4.014989 | 0.015651 |
| Glucose 6-phosphate | 2.691394 | 0.01192 |  |  |  |  |  | N-Acetylserine | 2.495035 | 0.024861 |
| Glycerol 3-phosphate | 1.962024 | 0.019354 |  |  |  |  |  | N-Acetyltaurine | 2.166863 | 0.046071 |
| Glycerophosphoethanolamine | 1.705073 | 0.019354 |  |  |  |  |  | N-Acetylthreonine | 2.143736 | 0.025696 |
| Glycerophosphorylcholine (GPC) | 2.118767 | 0.007703 |  |  |  |  |  | Nicotinamide | 2.140578 | 0.020694 |
| Hypoxanthine | 1.275325 | 0.015651 |  |  |  |  |  | Nicotinate | 2.442027 | 0.018845 |
| Inosine | 3.595295 | 0.020694 |  |  |  |  |  | Phenylpyruvate | 2.844708 | 0.018845 |
| Isobar*_b_* | 2.661121 | 0.015651 |  |  |  |  |  | Phytosphingosine | 2.660675 | 0.006819 |
| Isoleucylglycine | 2.349246 | 0.01192 |  |  |  |  |  | Propionylcarnitine | 3.327797 | 0.039194 |
| Itaconate | 7.469048 | 0.04543 |  |  |  |  |  | Sedoheptulose-7-phosphate | 1.956156 | 0.034502 |
| Myristoyl dihydrosphingomyelin (d18:0/14:0) | 2.197089 | 0.007736 |  |  |  |  |  | Uridine diphosphate-N-acetylglucosamine | 3.230689 | 0.046071 |
| N-Acetylglutamine | 2.159595 | 0.018168 |  |  |  |  |  |  |  |  |
| N-Acetylserine | 2.699501 | 0.021693 |  |  |  |  |  |  |  |  |
| N-Acetyltaurine | 2.424153 | 0.013342 |  |  |  |  |  |  |  |  |
| N-Formylmethionine | 2.085077 | 0.044015 |  |  |  |  |  |  |  |  |
| N6,N6,N6-trimethyllysine | 4.40925 | 0.006819 |  |  |  |  |  |  |  |  |
| Phenylalanylglycine | 2.061056 | 0.011943 |  |  |  |  |  |  |  |  |
| Phenylpyruvate | 2.372501 | 0.007703 |  |  |  |  |  |  |  |  |
| Phytosphingosine | 2.413743 | 0.047007 |  |  |  |  |  |  |  |  |
| Propionylcarnitine | 4.549604 | 0.020694 |  |  |  |  |  |  |  |  |
| Ribose | 2.173515 | 0.018347 |  |  |  |  |  |  |  |  |
| Thymine | 3.100301 | 0.039194 |  |  |  |  |  |  |  |  |
| Tyrosylglycine | 1.394609 | 0.015651 |  |  |  |  |  |  |  |  |
| Uridine diphosphate -N-acetylglucosamine | 4.099357 | 0.010261 |  |  |  |  |  |  |  |  |
| Uridine | 1.631586 | 0.018845 |  |  |  |  |  |  |  |  |

***_a_* fc: fold change, *_b_* Isobar: fructose 1,6-diphosphate, glucose 1,6-diphosphate, myo-inositol 1,4 or 1,3-diphosphate**
